# Removal of White Mineral Trioxide Aggregate Cement: A Promising Approach

**DOI:** 10.1155/2013/469164

**Published:** 2013-09-08

**Authors:** Mohammad Ali Saghiri, Franklin Garcia-Godoy, James L. Gutmann, Nader Sheibani, Armen Asatourian, Mehrdad Lotfi, Mayam Elyasi

**Affiliations:** ^1^Department of Ophthalmology and Visual Sciences, University of Wisconsin School of Medicine and Public Health, Madison, WI, USA; ^2^Bioscience Research Center, College of Dentistry, University of Tennessee Health Science Center, Memphis, TN, USA; ^3^Department of Restorative Sciences, Baylor College of Dentistry, Texas A&M University System Health Science Center, Dallas, TX, USA; ^4^Kamal Asgar Research Center (KARC) and Dental School, Tehran, Iran; ^5^Research Center for Pharmaceutical Nanotechnology and Department of Endodontics, Dental Faculty, Tabriz University (Medical Sciences), Tabriz, Iran; ^6^Private practice, Tehran, Iran

## Abstract

Removal of MTA from dentin by applying 37% hydrochloric acid (HCl) to reduce microhardness and push-out bond strength. Forty dentin slices were filled with WMTA and divided into two groups (*n* = 20). Ten slices remained untreated while others were exposed to either HCl or phosphate buffer saline (PBS) and all samples were subjected to pushout test. The mode of bond failures was determined by SEM analysis. Later, twenty glass tubes were filled with WMTA and divided into two groups (*n* = 10). One side of tube was exposed to HCl or PBS while the other side remained untreated and the microhardness was analyzed by testing machine. HCl showed significantly lower pushout strength and microhardness values (*P* = 0.0001), (*P* = 0.0001). HCl treated samples showed mixed bond failures dominantly, while PBS samples mostly showed adhesive failures. The results of this study can suggest the 37% HCl as an effective solution to aid the removal of MTA from the dentin surfaces.

## 1. Introduction 

Previous investigations have discussed the advantages of mineral trioxide aggregate (MTA) as a bioactive [1], biocompatible [2, 3], and radiopaque [4] root-end filling material which is capable of preventing microleakage [5, 6] and also having antibacterial efficiency [7]. One of the important characteristics of MTA is its unique sealing ability which gives it a widespread popularity [7]. The sealing ability is essential to prevent the leakage of microorganisms and their by-products which can lead to the failure of treatment [5, 8]. The undisputed retention characteristic of MTA is mostly attributed to the chemical bond between MTA surface and dentin wall [9]. Furthermore, the material's hydration phases can enhance the strength of this cement as well [10]. 

With respect to this, many authors have investigated the behavior of MTA in different environments to evaluate the solubility of this cement [4, 11–14]. Most of these investigators acclaimed that MTA has very low or even no solubility [4, 12–14]. Other investigators mentioned increased solubility through their long-term studies [15]. In addition, MTA was especially tested in low pH environments in many studies and authors found out that low pH values might affect the tensile strength [14], surface hardness [16], push-out bond strength to dentin surface [17], and even the sealing ability of this cement [18]. The impaired sealing ability of MTA was explained by the increase of porosities and voids occurring due to the acidic environment [17, 18]. This damage to the structure of cement is due to acidic corrosion which happens as a result of decomposition of calcium hydroxide and calcium sulfoaluminate phases [19, 20]. 

International Organization for Standardization (ISO 6876 standard) [21] and the American Dental Association (ADA) number 30 [22] have provided specifications for the assessment of dental root canal sealing materials. These specifications were used by previously mentioned studies to evaluate the solubility of MTA by determining the weight of cement loss in different environments [13, 15]. In another study, it was indicated that the thickness of MTA directly affects its displacement when it is used as an apical barrier. These authors showed that 4 mm thickness of MTA cement is more resistance to displacement than 1 mm thickness [23].

According to these facts, it can be presumed that MTA might lose its sealing ability and effective barrier thickness due to increasing solubility in the long term [15, 23]. This event might face the clinician with refreshment or even exchange previously applied MTA with new mixed cement in order to reestablish the sealing ability of this material. This issue is also of high importance in case of teeth which are treated by MTA and are subjected to endodontic retreatment. Previously, some authors have studied the removal of MTA from dentin surface by utilizing rotary endodontic files and ultrasonic devices [24]. Other investigators have tried to achieve this goal by using carbonic acid and mentioned that it could reduce the surface hardness of set WMTA remarkably [25].

The present study includes a pilot study to select the type of solution which can be used for the removal of WMTA cement. In the second part, the main study has been performed to evaluate the effect of hydrochloric acid solution on WMTA cement which was applied to dentin. The hypothesis tested was whether hydrochloric acid solution can decrease the microhardness and/or dislodgement force of set WMTA cement. This issue can make it easy to remove the set material from dentinal surface.

## 2. Materials and Methods

### 2.1. Pilot Study

First of all a pilot study was performed to conduct and select an effective solution: white ordinary Portland cement was mixed with distillated water with 3 : 1 powder/liquid ratio and packed into 20 cylindrical glass tubes with a 8 mm inner diameter and 10 mm length separately, and all samples were stored in incubator 37°C and 95 percentage humidity for 3 days. After incubation, samples were divided into four groups of 5 tubes in each (*n* = 5). Before exposure to solutions, one side of Portland cements tubes in each group was polished and cleaned gently to be ready for microhardness test. The Vickers microhardness test of each specimen was performed using a Clemex CMT surface hardness tester (Clemex Technologies Inc. Longueuil, Canada). After testing, the other sides of tubes in group A to D were exposed either to the vinegar (pH = 3.5), 37% hydrochloric acid (pH = 1.8), 5.25% Sodium hypochlorite (pH = 11.3), or phosphate buffer saline (pH = 7.2) for 60 seconds. Since exposure, samples were rinsed by distilled water for 1 minute, dried with paper point (Mani, Utsunomiya, Japan), and again polished and cleaned gently for microhardness test subsequently. Three indentations were made on the polished surface. The diagonal of the resulting indentation was measured under the microscope and the Vickers microhardness was calculated. The mean value of the hardness was used as the hardness value for each specimen. During the microhardness tests, after six indentations were made on surfaces, testing machines were calibrated by standard reference material (SRM) blocks which had been calibrated by euro productions calibration laboratory. The SRM block was cleaned with ethylene alcohol and soft wipe material and two indentations were made on it; then the diameter was measured and machine was adjusted. Differences between the means were analyzed by one-way ANOVA and post hoc Tukey's tests at 0.05 significance level.

### 2.2. Main Study

After pilot study, forty extracted single-rooted human teeth were used for this study. Samples were decoronated and sectioned horizontally at the midroot parts into 1.5 mm dentin slices. The canal spaces of the root slices were instrumented by number 2 through number 5 Gates-Glidden burs (Mani, Utsunomiya, Japan) to form 1.3 mm diameter standardized cavities. The specimens were then randomly divided into two groups (*n* = 20). In both groups, White ProRoot MTA (WMTA) (Dentsply Tulsa Dental, Tulsa, OK, USA) was mixed according to the manufacturer's instructions and placed inside the canal spaces of all root slices. Saline-moistened Gelatamp (Roeko-Colte'ne/Whaledent, Langenau, Germany) was used as a matrix while excess material was trimmed from the surface of the specimens with a scalpel and all samples were stored in incubator 37°C and 95 percentage humidity for three days. In group A, ten dentin slices were exposed to two drops of 37% hydrochloric acid (pH = 1.8) for 60 seconds and in group B, the specimens were exposed to phosphate buffered saline similar to serve as control group. Other ten samples of groups A and B remained without any treatment and serve as control for treated samples.

### 2.3. Push-Out Test

The push-out bond strengths were measured by using Zwick/Roell Z050 universal testing machine (Ulm, Germany). The WMTA was loaded with a 0.7 mm diameter cylindrical stainless steel plunger at a speed of 1. The maximum load applied to the WMTA was recorded in Newton before the occurrence of dislodgement. To express the bond strength in MPa, the recorded value in Newtons was divided by area in mm^2^ calculated by the following formula: 2*πr* × *h*, where *π* is the constant 3.14, *r* is the root canal radius, and *h* is the thickness of the root slice in millimeters. The slices were then examined under scanning electron microscope (SEM) at ×40 magnification to determine the mode of the bond failure. The samples failure mode was determined according to following classification.

 
*Adhesive failure* occurred at the WMTA and dentin interface.
 
*Cohesive failure* occurred within the WMTA cement.
 
*Mixed failure* occurred both at the interface and within WMTA cement.

The data were analyzed by using one-way analysis of variance (ANOVA), and Tukey's post hoc tests.

### 2.4. Microhardness

Similar to pilot study, twenty glass cylindrical tubes were used for this part of study. WMTA was mixed with distilled water at 0.3 mL/g liquid-to-powder ratio. The mixed cement was packed into the tubes using a nonsurgical manual MTA carrier (Dentsply Tulsa Dental, Tulsa, OK, USA) and hand pressure [26, 27]. Samples were incubated at 37°C and 95% humidity for 3 days. After incubation, WMTA tubes were divided into two groups of 10 tubes in each (*n* = 10). Before exposure to solutions, one side of WMTA tubes in each group was polished and cleaned gently to make ready for microhardness test. After testing, the other sides of tubes in groups A were exposed to 37% hydrochloric acid (pH = 1.8), while in group B samples were treated by phosphate buffered saline (pH = 7.2) as control group for 60 seconds. Since exposure, samples were rinsed by distilled water, dried with paper point (Mani, Utsunomiya, Japan), and again polished and cleaned gently for Microhardness test subsequently. A two-way ANOVA was conducted that examined the effect of material and pH on microhardness. Our dependent variable, microhardness, was normally distributed for the groups formed by the combination of the material and pH as assessed by the kolmogorov-Smirnov test. 

### 2.5. SEM Analysis

Five Samples from groups A and B were chosen randomly and underwent scanning electronic microscopy (SEM) examination. After the dislodgment of MTA, samples of groups A and B were irrigated with 10 mL of distilled water and vertically grooved on the buccal and lingual surfaces with a diamond disc without entering the canals and split longitudinally with a chisel. One half of each sample was randomly chosen, placed in 2% glutaraldehyde for 24 hours, and then rinsed 3 times with sodium cacodylate buffered solution (0.1 M, pH = 7.2). All samples were dehydrated with ascending concentrations of ethyl alcohol (30–100%), placed in a desiccators for 24 hours and mounted on a metallic stub. After coating the samples with gold, SEM micrographs were taken (Leo. 440i; Oxford Microscopy, Oxford, UK) (×500).

## 3. Results

### 3.1. Pilot Study

**Figure 1:**
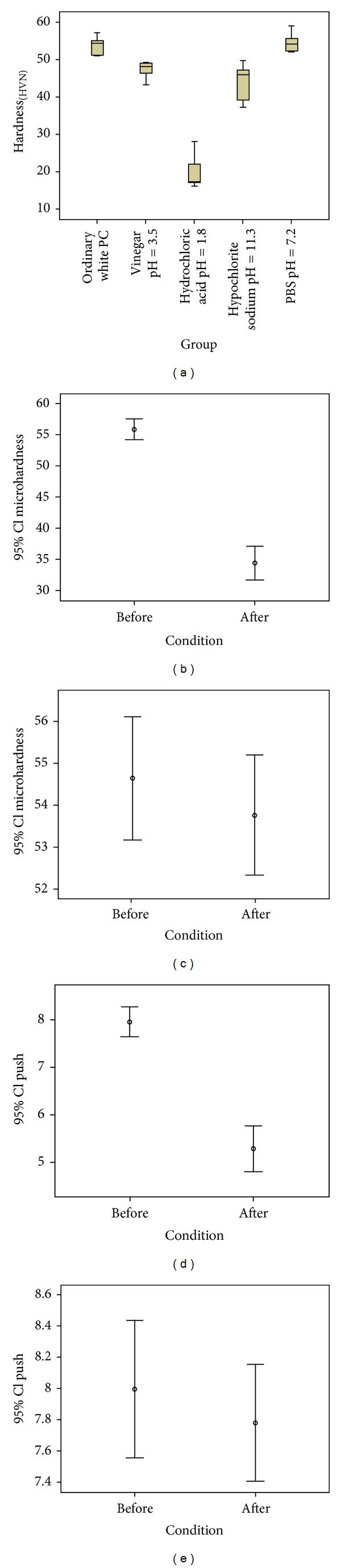
(a) Box plots of the means ± standard deviations of the surface microhardness of pilot study on white ordinary Portland cement. (b) Box plot of the means ± standard deviations of the surface microhardness (overall) before exposure to 37% hydrochloric acid and phosphate buffer saline. (c) Box plot of the means ± standard deviations of the surface microhardness (overall) after exposure to 37% hydrochloric acid and phosphate buffer saline. (d) Box plot of the means ± standard deviations of the push-out bond strength before exposure to 37% hydrochloric acid and phosphate buffer saline. (e) Box plot of the means ± standard deviations of the push-out bond strength after exposure of 37% hydrochloric acid and phosphate buffer saline which illustrate the means ± standard deviations.

The surface microhardness value of ordinary white Portland cement as control group and after exposure to Vinegar (pH = 3.5), 37% hydrochloric acid (pH = 1.8) Sodium Hypochlorite (pH = 11.3), PBS (pH = 7.2) were 53.76 ± 1.18, 47.22 ± 1.11, 20.18 ± 2.23, 43.88 ± 2.41, and 54.69 ± 1.28, respectively (Figure 1(a)). Post hoc Tukey's test revealed significant differences among the 37% hydrochloric acid, sodium hypochlorite, and control group (*P* < 0.001). There was no significant difference between control and phosphate buffer saline groups (*P* = 0.995).

### 3.2. Main Study

#### 3.2.1. Microhardness

The means ± standard deviations of microhardness values for WMTA before and after applying hydrochloric acid were 55.9 ± 2.32 and 34.45 ± 3.77 (VHN), respectively. There was homogeneity of variance as assessed by Levene's Test for Equality of Variances. Therefore, an independent *t*-test was run on the data as well as 95% confidence intervals (CI) for the mean difference. It was found that, after applying 37% hydrochloric acid on WMTA, microhardness was significantly reduced (*P* = 0.0001) (Figure 1(b)). However, the means ± standard deviations of microhardness for WMTA before and after applying normal saline were 54.63 ± 2.305 and 53.75 ± 2.00, respectively. It means that applying normal saline on the WMTA could not significantly affect the microhardness value of the material (*P* = 0.34) (Figure 1(c)).

#### 3.2.2. Push-Out Test

The means ± standard deviations of push-out strength for WMTA before and after applying hydrochloric acid were 7.94 ± 0.44 and 5.27 ± 0.65, respectively. There was homogeneity of variance as assessed by Levene's Test for Equality of Variances. Therefore, an independent *t*-test was run on the data as well as 95% confidence intervals (CI) for the mean difference. It was found that after applying 37% hydrochloric acid on WMTA, push-out strength was significantly reduced (*P* = 0.0001) (Figure 1(d)). However, the means ± standard deviations of push-out for WMTA before and after applying PBS were 7.99 ± 0.61 and 7.78 ± 0.52, respectively. It means that applying normal saline on WMTA could not significantly affect the push bond strength value of the material (*P* = 0.41) (Figure 1(e)). 

#### 3.2.3. SEM Results

**Figure 2:**
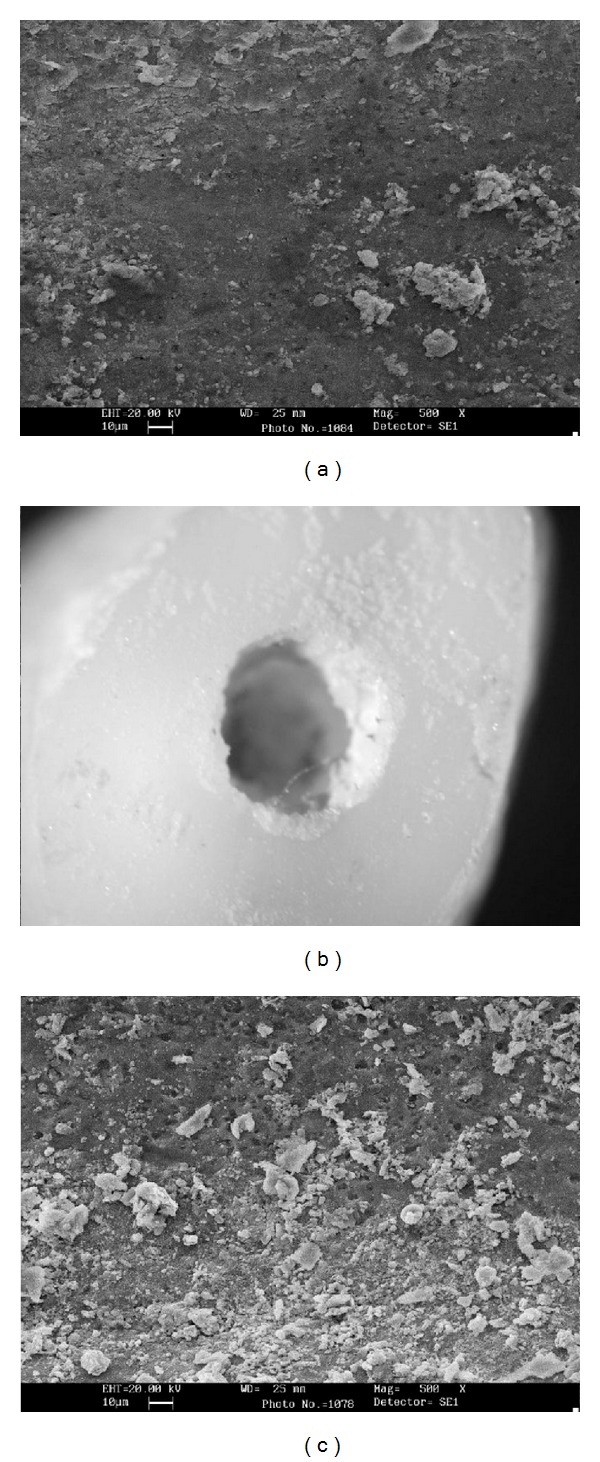
Mode of failures: (a) adhesive failure; note the clean canal wall ×500. (b) A sample of mixed failure within cement (adhesive and Cohesive) ×40. (c) cohesive failure within cement ×500.

The analysis of SEM images has revealed that the mode of bond failure in samples of PBS group was adhesive type dominantly, while in 37% HCL group it was mostly mixed failure (Figure 2).

## 4. Discussion

The removal of MTA cement from dentinal wall is a newly introduced concept which was discussed by other authors previously as well [24, 25]. These investigators, since using mechanical devices such as NiTi rotary files and ultrasonic instruments, did not report any significant results in removal of MTA from dentinal surface. Previously, it was mentioned that acidic environment can affect the sealing ability of this cement [18] and finally required a clinician to refresh applied cement in order to reestablish the impaired sealing properties of MTA in low pH value situations [18]. As in the literature, there was no single method declared to check the removal ability of WMTA, so we selected hardness property to examine in our study, which was used by another previous study as well [25]. Authors have presumed that by decreasing the microhardness of MTA it could be removed more easily than hard and set cement. According to previous studies, many factors such as low pH value of the environment, less humidity, and chelating agents might adversely affect the MTA microhardness [28]. Push-out test is another test considered in the main part which is selected to test whether acidic solution is able to decrease the dislodgment force of MTA. 

In the present study, authors have selected some chemical solutions to analyze their ability on the removal of MTA. In the industry, hydrochloric acid has been introduced for the removal of cement. It is mentioned that the removal of cement after use is readily accomplished by passing acids such as dilute hydrochloric acid in contact with cement until it dissolves or decomposes [29]. Another acidic material tested is acetic acid which was discussed by some authors as well. These investigators mentioned that acetic acid with pH value of 3 is able to make corrosion in the structure of Portland cement [30]. These choices were made according to previous investigations where mentioned acidic environments can affect the physical properties of MTA such as surface hardness and push-out bond strength [16, 17]. This issue was also discussed by other investigators who pointed out that acidic substances can make disintegration in the structure of MTA due to its alkaline nature [25]. Sodium hypochlorite is another solution which has been shown to be effective on MTA by some authors [31]. They reminded that 5.25% sodium hypochlorite can significantly decrease the microhardness of MTA cement [31] which is a reason for using this substance in our pilot study. 

The results of pilot study showed that acid solutions are able to reduce the microhardness of cement significantly. Similar results were indicated by previous authors, which showed that low pH value solutions can significantly decrease the surface hardness of Portland-based cements [28]. This outcome is in consistent with a previously done study, where authors have mentioned that carbonic acid could significantly reduce surface hardness of MTA [25]. However, 37% hydrochloric acid could reduce surface hardness more than acetic acid which can be explained by the lower pH value of hydrochloric acid (pH = 1.8) in comparison with acetic acid (pH = 3.5). Also a 5.25% Sodium hypochlorite significantly reduced surface harness which is similar to the results of previously mentioned study [31].

Due to the results of microhardness tests in the pilot part of study, authors have concluded that 37% hydrochloric acid with pH value of 1.8 is more effective than other tested materials in order to loosen Portland cement structure. Although previous investigators have mentioned that carbonic acid, as a weak acidic solution, was successfully able to dissolve set MTA [25], these authors have only evaluated the surface hardness while in the present study authors have used push-out bond strength and microhardness of set MTA in order to measure the effect 37% hydrochloric acid on the deeper layers of MTA which might resulted in removal of set material much more thoroughly than the surface layers solely. In previous studies many investigators mentioned that the push-out bond strength of MTA-dentin surface can significantly decreased when MTA was exposed to acidic solutions with low pH values [17, 18]. Results of the present study showed that 37% hydrochloric acid is significantly able to decrease dislodgement force of MTA in comparison with PBS, which is consistent with other previously mentioned studies.

In microhardness evaluations of MTA after being exposed to hydrochloric acid it was noted that this solution can significantly decrease the microhardness of MTA which is similar to findings of other investigators [16, 21]. During the contact of acidic solutions with MTA, structural changes can occur which resulted in large voids and porosities inside the mixed cement. These voids are mainly because of decomposition of calcium hydroxide, C–S–H, and the calcium sulfoaluminate phases [19]. SEM analysis of samples that underwent push-out test indicated the failure modes of MTA-dentin surface. In samples treated with 37% hydrochloric acid the bond failures were mixed cohesive and adhesive while in PBS exposed samples in most of the cases were adhesive failures. This difference in the types of bond failure made by hydrochloric acid and PBS can be explained by the changes which were induced by acidic solutions. Acidic materials can penetrate into the cement structure through water content of MTA after hydration phase of cement and by decomposing the cement microstructure [19] cohesive bond failure can be resulted finally. These changes have not been seen in samples treated by PBS. 

## 5. Conclusions

In accordance with the results of the pilot and main studies, authors have made the following conclusions.

1. Removing MTA from dentinal surface is of concern for clinicians especially in cases which refreshment of MTA or retreatment is required. To accomplish this, low pH value of chemical solutions can be used to decrease the properties of MTA such as microhardness and dislodgement resistance.
2. Among tested solutions, 37% hydrochloric acid can be regarded as a powerful substance which can successfully lower the microhardness and push-out strength of MTA. The significant reduction in the properties of MTA can aid better removal of MTA from dentinal surface.

As an eye to the future, it should be mentioned that the present study was a preliminary study which was done to introduce an effective solution in WMTA cement removal. Definitely, future studies are needed to evaluate the biocompatibility and the possible side effects of this solution on dentin. Also, future investigations can address to introduce a suitable method for application of this solution to WMTA cement, while it has the minimal contact to dentinal surface.
